# Supplementary material for: Accessible interactive learning of mathematical expressions for school students with visual disabilities
Source: PeerJ Comput Sci. 2024 Dec 23;10:e2599. doi: 10.7717/peerj-cs.2599 (PMC11784810; doi:10.7717/peerj-cs.2599)
Supplement: Supplemental Information 1 [file peerj-cs-10-2599-s001.docx]

**Student Feedback Questionnaire**

**Dear Student,**

We appreciate your participation in this survey. Your feedback is crucial in assessing the effectiveness of a new solution designed to aid in learning mathematics. Please indicate the extent to which you agree or disagree with each statement below.

**Instructions:** For each statement, please select one of the following options: Strongly Agree, Agree, Neutral, Disagree, or Strongly Disagree.

1. **I found that the solution assisted me in learning mathematics.**
   - Strongly Agree
   - Agree
   - Neutral
   - Disagree
   - Strongly Disagree
2. **Compared with Access8Math and screen reader, the proposed solution comprehends math resources and navigates and identifies structural elements of mathematical expressions better.**
   - Strongly Agree
   - Agree
   - Neutral
   - Disagree
   - Strongly Disagree
3. **I found that using this solution has shortened the time required to learn math.**
   - Strongly Agree
   - Agree
   - Neutral
   - Disagree
   - Strongly Disagree
4. **I would consider this solution to be beneficial.**
   - Strongly Agree
   - Agree
   - Neutral
   - Disagree
   - Strongly Disagree
5. **I found that this solution is easy to use.**
   - Strongly Agree
   - Agree
   - Neutral
   - Disagree
   - Strongly Disagree
6. **I found that this solution enabled me to concentrate better while studying.**
   - Strongly Agree
   - Agree
   - Neutral
   - Disagree
   - Strongly Disagree
7. **This solution would be useful to me in studying math, and I would recommend it to others.**
   - Strongly Agree
   - Agree
   - Neutral
   - Disagree
   - Strongly Disagree

**Thank you for your time and valuable feedback!**
