# Supplementary material for: Accessible interactive learning of mathematical expressions for school students with visual disabilities
Source: PeerJ Comput Sci. 2024 Dec 23;10:e2599. doi: 10.7717/peerj-cs.2599 (PMC11784810; doi:10.7717/peerj-cs.2599)
Supplement: Supplemental Information 2 [file peerj-cs-10-2599-s002.docx]

**Research Questionnaire**

**Dear Faculty Member,**

We appreciate your participation in this survey. Your responses will help us evaluate the effectiveness of a new solution designed to aid students in comprehending math handouts, exercises, and exams. Please indicate the extent to which you agree or disagree with each statement below.

**Instructions:** For each statement, please select one of the following options: Strongly Agree, Agree, Neutral, Disagree, or Strongly Disagree.

1. **I discovered that this solution aided my students in comprehending math handouts, exercises, and exams.**
   - Strongly Agree
   - Agree
   - Neutral
   - Disagree
   - Strongly Disagree
2. **I believe that this solution could assist my students in focusing more on their math studies.**
   - Strongly Agree
   - Agree
   - Neutral
   - Disagree
   - Strongly Disagree
3. **I have found that this solution is particularly useful when solving math problems.**
   - Strongly Agree
   - Agree
   - Neutral
   - Disagree
   - Strongly Disagree
4. **I think that this solution has the potential to improve my students' capacity for independent study.**
   - Strongly Agree
   - Agree
   - Neutral
   - Disagree
   - Strongly Disagree
5. **In my experience, this solution is user-friendly.**
   - Strongly Agree
   - Agree
   - Neutral
   - Disagree
   - Strongly Disagree
6. **I found that this solution navigates and identifies all elements of mathematical expressions comparatively better than screen readers and Access8Math.**
   - Strongly Agree
   - Agree
   - Neutral
   - Disagree
   - Strongly Disagree
7. **I wholeheartedly endorse this solution and would recommend it to others.**
   - Strongly Agree
   - Agree
   - Neutral
   - Disagree
   - Strongly Disagree

**Thank you for your time and valuable feedback!**
