# Supplementary material for: Accessible interactive learning of mathematical expressions for school students with visual disabilities
Source: PeerJ Comput Sci. 2024 Dec 23;10:e2599. doi: 10.7717/peerj-cs.2599 (PMC11784810; doi:10.7717/peerj-cs.2599)
Supplement: Supplemental Information 5 [file peerj-cs-10-2599-s005.docx]

**Source File: Students and Instructors feedback**

**Introduction**

The raw feedback we collected from instructors and students about their experience with the proposed educational tool is presented here. The feedback is categorized as themes with a direct quote to refer to.

**1. Participant Demographics**

**Instructors:**

- Total: 15 (8 Male, 7 Female)
- Locations: Peshawar, Abbottabad, Swat (Government Institutes for the Blind)

**Students:**

- Total: 94 (50 Male, 44 Female)
- Locations: Peshawar, Abbottabad, Swat (Government Institutes for the Blind)

**2. Raw Feedback**

**Theme 1: Ease of Use**

- **Instructors:**
  - *"The navigation was straightforward, and the students could use it with minimal guidance."* (Male, Peshawar)
  - *"The interface was simple and well-organized, which helped us save time during lessons."* (Female, Abbottabad)
- **Students:**
  - *"I found it easy to use, even though I’m not very familiar with technology."* (Male, Swat)
  - *"Accessing lessons was simple, but I needed help at the start."* (Female, Abbottabad)

**Theme 2: Improved Focus and Comprehension**

- **Instructors:**
  - *"* *This solution provides a complete learning experience by integrating navigation and analysis of math expressions, something the other tools don’t offer in the context of mathematical learning."* (Female, Swat)
  - *"I observed a positive change in how students interacted with the content."* (Male, Abbottabad)
- **Students:**
  - *"* *The proposed goes beyond just reading; it helps me navigate, understand, and analyze mathematical expressions, which makes learning math much easier compared to NVDA and Access8Math.* (Male, Peshawar)
  - *"The interactivity made it more interesting, and I could focus more during lessons."* (Female, Swat)

**Theme 3: Areas for Improvement**

- **Instructors:**
  - *"It took some time to get used to the new tool, but once we did, it was very effective."* (Male, Peshawar)
  - *"Providing a training session would make the transition smoother for both teachers and students."* (Female, Abbottabad)
- **Students:**
  - *"The change from the old system was a bit confusing at first."* (Male, Swat)
  - *"Some features need better explanation or more practice before we can use them effectively."* (Female, Peshawar)

**3. Categorization of Feedback**

| **Category** | **Instructor Comments** | **Student Comments** |
| --- | --- | --- |
| **Ease of Use** | - "Straightforward navigation." | - "Easy to use but needed initial help." |
| **Improved Focus** | - "Students engaged more and understood better." | - "Content was easier to grasp, thanks to interactivity." |
| **Areas for Improvement** | - "Initial transition was difficult; training would help." | - "Some features were confusing without guidance." |

**4. Implications and Recommendations**

- Training sessions or onboarding materials (e.g, tutorials) for users.
- Enhanced tool documentation with step-by-step guides.
- Additional practice or support to ease the transition to the new system.
